# A Shift Toward Building with Nature in the Dredging and Port Development Industries: Managerial Implications for Projects in or Near Natura 2000 Areas

**DOI:** 10.1007/s00267-014-0285-z

**Published:** 2014-05-09

**Authors:** Vera Vikolainen, Hans Bressers, Kris Lulofs

**Affiliations:** Twente Centre for Studies of Technology and Sustainable Development (CSTM), University of Twente, Postbus 217, 7500 AE Enschede, The Netherlands

**Keywords:** Dredging, Port development, Building with Nature, Natura 2000, Project management, North-West Europe

## Abstract

*Building with Nature* is a new approach to designing water infrastructure, one that seeks to realize socioeconomic project goals in harmony with the environment. The Dutch dredging industry is promoting its application in the Netherlands, but similar concepts are emerging internationally. The *Working with Nature* concept has been developed under the auspices of the World Association for Waterborne Transport Infrastructure, *Engineering with Nature* by the US Army Corps of Engineers, and *Flanders Bays 2100* by a group of Belgian dredging companies and international consultants. The research discussed in this article focuses on the feasibility of implementing the *Building with Nature* approach in the context of EU Natura 2000 governance. The initial expectation of the industry was that Natura 2000 regulations would obstruct innovative *Building with Nature* attempts. The empirical evidence points to a shift toward *Building with Nature* have taken place on the governance and project levels, and the goals of Natura 2000 and *Building with Nature* converging in practice. Using specific project-level variables identified by researchers, guidance for project development in Natura 2000 areas was proposed. We conclude by discussing the implications of the research results for the dredging industry dealing with Natura 2000 regulations in Europe and similar overarching nature regulations elsewhere.

## Introduction

The dredging industry is searching for ways to make their operations more environmentally friendly, such as by responsible disposal of dredged material (Köthe and de Boer 2003, Mink 2005, 2006, International Association of Dredging Companies 2012), by introducing an ecosystem approach to dredging (Mink 2008), and by using nature and natural processes as a starting point in project design and development (International Association of Dredging Companies 2010). The initial expectation was that opportunities for realizing creative and innovative ideas for improving environmental conditions would be limited as a consequence of the existing environmental legislation (European Dredging Association 2007). In particular, the EU nature conservation policy was seen as putting pressure on economic activities in estuaries and coasts and leading to conflict with port-related activities that involve dredging. Several reviews of project histories (van Hooydonk 2006, Mink 2007) found that ports, maritime, and coastal infrastructures are often close to protected nature areas and appear to be especially affected by the provisions of the EU nature conservation policy, such as the Birds and Habitats Directives. These provisions caused frequent delays in project execution were unclear, and left room for diverging interpretations, which an ever-growing case law failed to clarify. In many cases, the port authority’s opinion had no influence on the designation of a site as a valuable nature area, and the planned future use of an expanded facility had not been taken into consideration. If a planned expansion ultimately went according to plan, the additional costs for procedural matters, for environmental damage compensation, and for resulting delays, fell entirely on the project developer (Mink et al. 2007). The EU Commission’s DG Environment, supported by NGOs, responded by saying that the Directives were misunderstood and, if well used, could be a positive element in economic development (Schmedtje and Kremer 2008).

The industry’s growing environmental consciousness as well as the increasing pressure of environmental legislation on port development and dredging have prompted the emergence of new approaches to designing water infrastructure. One of the approaches, which seeks innovative project designs that realize socioeconomic project goals in harmony with the environment, is *Building with Nature*. *Building with Nature* was introduced by the Czech hydraulic engineer Svašek in 1979 and was further explored and linked to the field of coastal management by Waterman (2008, 2010). Practitioners hoped, by integrating environmental concerns as early as the project design stage, which they would better serve the environment and society and improve project implementation in the field of coastal and delta infrastructure. Sector organizations and supranational authorities have recently adopted *Building with Nature*, albeit under slightly different names such as *Working with Nature* by The World Association for Waterborne Transport Infrastructure (PIANC 2011). The approach has been recommended by the European Commission for port development and dredging (European Commission 2011), inland waterway development (European Commission 2012), and has taken root in Dutch water management (Delta Commission 2008). Consequently, the relevance of the *Building with Nature* concept for water infrastructure development in estuaries and coasts is expected to increase in the coming years. In the Netherlands, scientific research into the application of *Building with Nature* took place between 2008 and 2012 under the auspices of the EcoShape Foundation (EcoShape 2012). In this article, we present the empirical results of one of the research projects within that research program, related to the feasibility of applying *Building with Nature* in the context of EU Natura 2000 governance.

The research question addressed in this article is: what are the implications of the European nature conservation policy for project developers intending to apply *Building with Nature* in or near Natura 2000 areas? In answering the question, the provisions of the Birds and Habitats Directives, which form the cornerstone of the EU’s Natura 2000, are first summarized. Following this, the origins of the *Building with Nature* approach are outlined and a definition given. Thereafter, the empirical research results are presented and the learning process that took place on three governance levels is explained. Project-level explanatory variables originating from the research are presented and then used to elaborate guidance for the application of *Building with Nature* in Natura 2000 areas. The article concludes by discussing the implications of the research results for the dredging industry in Europe and other continents.

## Natura 2000

Many ports in North-West Europe are situated at the mouth of estuaries or along the coastline. Besides being among the most densely populated areas in the world, estuaries and coastal zones are also among the most dynamic and complex ecosystems, made up of sandbanks, mudflats, salt marshes, sand dunes, coastal lagoons, shallow inlets and bays, reefs, islets and small islands, sandy beaches, and sea cliffs. In the EU, these valuable ecosystems are protected by the Birds and Habitats Directives (EEC 1979, 1992).

These directives form the legal basis for the Natura 2000 biodiversity network. EU Member States are required to designate Special Areas of Conservation (SAC) under the Habitats Directive and Special Protection Areas (SPA) under the Birds Directive. These areas together make up the EU-wide Biodiversity Network: Natura 2000. Member States are required to assess the conservation status and establish conservation objectives for the species and habitats in these areas. Article 6 of the Habitats Directive introduced a requirement for a thorough assessment of any plan or project that was likely to have a significant effect on the integrity of a Natura 2000 site, known as a Habitat assessment. This assessment obliges the authorities to evaluate whether a plan or project is likely to have significant effects on a Natura 2000 site and, if that is the case, to carry out an appropriate assessment of these effects (Article 6 paragraph 3). In the event of a negative assessment, the authorities must consider possible alternatives and, if there are none, state the imperative reasons for overriding public interest and further take compensatory measures (Article 6 paragraph 4).

Port developments and economic activities in general regularly conflict with the desire to conserve estuarine and coastal habitats (Mink et al. 2007). The question as to whether there is a possibility of significant effects has been a stumbling block for many plans and projects. In many instances, authorities played down the likely significance of effects and/or failed to carry out an appropriate assessment. They were subsequently challenged by environmental NGOs in court, resulting in severe delays and the cancelation of many water infrastructure projects in North-West Europe (van Hooydonk 2006). The most prominent examples are the Antwerp and Rotterdam port extensions, each delayed for more than a year.

## Building with Nature

*Building with Nature* seeks to develop new ways of thinking and acting in relation to sustainable coastal development. This €30 million program was initiated by the Dutch dredging industry, with partners representing academia, research institutes, consultancies, and public parties. The aim of the program was to seek infrastructure solutions that both utilize and enhance the natural system, such that the ecological and economic interests of a project are mutually reinforcing. In addition to the research presented in this article, the societal component of the *Building with Nature* program included the feasibility of local decision-making arenas (Smit 2011), the role of knowledge, and uncertainty in decision-making (van den Hoek et al. 2012, van den Hoek 2014, Janssen et al. 2014), and innovative project arrangements (Korbee and van Tatenhove 2013, Korbee et al. 2014). It was launched in 2008 by the EcoShape Foundation as a public–private innovation program. The first phase ran until 2012 (EcoShape 2012), and it is currently in its second phase. The Foundation considers water infrastructure to be designed according to *Building with Nature* principles if its design fulfills the following criteria (Waterman 2008, 2010, Aarninkhof et al. 2010, EcoShape 2012):

1. It explores opportunities for nature development during the initial project design stage and integrates socioeconomic and ecological goals (*integration* of nature).
2. It uses natural dynamics and materials that occur in nature in the context of hydrological and morphological situations to achieve the project’s goals (*use of* natural dynamics).
3. It creates opportunities for the development of new nature and improves the ecological values currently present in the project area (*improvement* of nature).

The EcoShape Foundation has applied the *Building with Nature* approach to large-scale replenishment work involving some 20 million m^3^ of sand along the Dutch coast (Aarninkhof et al. 2010, van Slobbe and Lulofs 2011, van den Hoek et al. 2012, van Slobbe et al. 2013, Stive et al. 2013).

In parallel with *Building with Nature*, the *Working with Nature* concept was developed under the auspices of the World Association for Waterborne Transport Infrastructure (PIANC 2011), *Engineering with Nature* by the US Army Corps of Engineers (Bridges and Walker 2011), and *Flanders Bays 2100* by a group of Belgian dredging companies and international consultants (International Association of Dredging Companies, 2010). All these concepts are similar in that they attempt to reconcile tensions between socioeconomic and ecological goals in water infrastructure projects. This article will focus on the *Building with Nature* approach as defined above.

Based on the negative experiences during regular dredging operations in Natura 2000 areas, the EcoShape Foundation expected the Natura 2000 requirements to hinder *Building with Nature* projects just as they had hindered projects in the past. Thus, the initial hypothesis of this research project was that the conservation-oriented Natura 2000 represented a regulatory bottleneck to innovative *Building with Nature* practices.

## Research Methods

The research reported in this article was carried out between 2008 and 2012 and was based on a small-N set of qualitative case studies (Yin 2003). Method triangulation (Webb et al. 1966, Denzin 1970, Meffert and Gschwend 2012) was adopted to minimize the danger that any relationship found between the presumed cause (the extent of designing in line with *Building with Nature* in a water infrastructure project) and the effect (the outcome of implementing Natura 2000 requirements at the project level) was a result of bias. As such, the researcher analyzed the presumed cause-effect relationship using three different case study designs: multiple (14 cases), quasi-experimental (2 cases), and longitudinal (1 case).

## Case Selection

The goal in case selection was to generate a subset of projects covering the full range of *Building with Nature* incorporation in their design from a larger population of water infrastructure projects in North-West European estuaries and coasts. Many such projects were identifiable, but enumeration of them all would be nearly impossible. The subset had to be sufficient for credible comparison of each case with all the others in the set (pairwise comparison). The cases were selected using the diverse case method (Seawright and Gerring, 2008). This method requires the full range of values characterizing the dependent and independent variables (X, Y) or their relationship (X/Y) to be included. The variables in this research were the extent of the design fulfilling *Building with Nature* expectations (X) and the implementation of Natura 2000 requirements in a project (Y). Subsequently, the 14 cases were selected so as to cover the full range of variation in the variables. In terms of cases with a high *Building with Nature* design content, the selection amounts to almost the entire population, as such projects are limited. Each component of the former (X), listed in Sect. 3, i.e., *integration*, *use of*, and *improvement* of nature was assigned a “+” if it was present in a project design; or a “0” if it was absent. The assessment of the components amounted to an informed judgment based on the research data and insider information provided by interviewees. The outcome of the implementation (Y) was measured in terms of the assessment of the project design’s effects according to the requirements of the Article 6 procedure. The outcome was deemed *successful* if a project design raised no objections or was successfully defended in court, whereas an *unsuccessful* project was one that raised objections leading to its design having to be reconsidered due to a court ruling for it to be implemented. The outcomes were assigned a ☺ or a ☹, respectively.

The subset of 14 water infrastructure projects was located in the Netherlands, in Belgium, in the UK, and in Germany. Dutch projects dominated the selection with six projects (compared to two in Belgium, three in Germany, two in the UK, and one Belgian/Dutch cross-border project) for reasons of accessibility and financial constraints. To minimize potential country bias, a spread in the ranges of the independent variables was ensured in the cases from a single country. A distinct advantage of diverse case selection, as pointed out by Seawright and Gerring (2008), is that it enhances the representativeness of the sample of cases chosen by the researcher. On the other hand, the inclusion of the full range of variation may distort the actual distribution of cases across the spectrum. However, the distribution of cases across the spectrum was not a primary concern in this research since we were more concerned with the relationship between the main variables of interest.

Three cases in this subset were selected for further analysis on the basis of expectations about their valuable information content. In two of these cases, the opposing implementation outcomes seemed to be strongly related to the distinct values of the independent variable, and these were selected for the quasi-experimental case study. In the other case, the independent variable appeared to demonstrate changes over a period of some 30 years and formed an ideal basis for our longitudinal case study. Information-oriented selection, as used here, is useful in maximizing the utility of information from small samples and single cases (Flyvbjerg 2006). Although the projects studied were necessarily unrepresentative of the wider population in terms of meeting the requirements of quantitative methodologies, they do, as advocated by (George and Bennet 2005), enable contingent generalizations for project subtypes similar to the cases studied.

### Data Collection

The main sources of the research data were qualitative semi-structured interviews and documents. The subset of 14 cases is built on data collected between 2008 and 2010, including interviews with informants from public and private organizations, project documentation supplied by the interviewees, and a study of historical cases available in the literature. Informants representing all the organizations actively involved were interviewed for each case.

Data on the three in-depth cases were collected in August and September 2009 and in March 2011. For these case studies, the data-gathering logic first involved gathering general information from the dedicated project websites. A general inquiry, stating the goals and purpose of the research, was then sent to the project secretariat or directly to a project manager. An initial list of involved government institutions, private, and non-profit stakeholders, and their corresponding roles in the implementation processes, was drawn up with the help of the project secretariat/project manager. Next, one respondent from each government institution and stakeholder organization was interviewed using qualitative semi-structured interviews. To ensure that all actors were covered, including project opponents, each respondent was asked to name all the participating actors, whose roles were also later crosschecked with document sources. To ensure that all the relevant documents were obtained, all respondents were asked to supply the documentation that they considered relevant for the project and/or mentioned during the interview. This snowballing approach should ensure that the sets of documents and respondents are complete. Research ethics were taken into account by informing the participants what their participation in the research would entail. Confidentiality and anonymity of the information supplied were guaranteed.

### Data Analysis

Qualitative data analysis techniques were applied in extracting the results. In analyzing the subset of 14 cases, a systematic assessment of the key variables was carried out. With the two cases selected for the quasi-experimental case study, a modus operandi method, also known as the “detective paradigm” (Scriven 1976), was applied. In the longitudinal case study, we carried out a theory-guided reconstruction of chronological events, a special form of time series analysis (Yin 2003, p.125).

## Empirical results

First, the 14 water infrastructure projects were assessed in terms of the two main variables: the application of Article 6 of the Habitats Directive and the respective project design (Vikolainen et al. 2011). A majority of projects that were successful in applying this Article had at least some features of the *Building with Nature* ideas included in their design (Table 1).

**Table 1:** The results from a subset of 14 cases. BwN = *Building with Nature*; Art. 6 = Outcome of Habitats Directive Assessment Article 6 (☺ successful; ☹ unsuccessful; ? unknown at the time of writing; source: Vikolainen 2012)

Art. 6:	☺	**?**	☹
BwN			
**+++**	•Delfland Coast (Netherlands);		
	•Oyster Reefs - Eastern Scheldt (Netherlands);		
	•Kruibeke-Bazel-Rupelmonde Flood Control Area (Belgium)		
**++**	•Humber Estuary: Hull and		
	Immingham Ports (UK);		
	•Bremerhaven Container Port CT4 (Germany);		
	•Flexible Dredging Strategy in the Western Scheldt (Netherlands);		
	•Coastal Zone Zeewolde (Netherlands)		
**+**			•Port of Rotterdam: Second Maasvlakte Extension (Netherlands);
			•Waterfront Harderwijk (Netherlands)
0	•Hamburg Airbus Facility Extension on the River Elbe (Germany)	•Hamburg Tidal Elbe and Fairway Deepening (Germany)	•Western Scheldt Container Terminal (Netherlands);
			•Port of Southampton Dibden Bay (UK);
			•Port of Antwerp: Deurganck Dock (Belgium)

Following this analysis, the three information-rich cases were selected from this subset for further analysis. Two Dutch projects, Waterfront Harderwijk and Coastal Zone Zeewolde, were selected, because they were similar in so many respects (location, type of project, the same local environmental NGO lodging an appeal, and on the same grounds), yet the implementation outcomes were diametrically opposed. As such, the cases were appropriate for a quasi-experimental comparison, and this made it possible to test the hypothesis that the extent of *Building with Nature* in the project design explained the opposite outcomes (Vikolainen et al. 2012). The analysis confirmed that the integration of nature and socioeconomic goals (the first component of *Building with Nature*) increased the likelihood of coastal zone development projects being approved if their fulfillment of Natura 2000 requirements is challenged in court. A longitudinal case study of the Kruibeke-Bazel-Rupelmonde flood control project in Belgium (Vikolainen et al. 2013a) showed a gradual progression in the project design from a pure engineering concept toward a *Building with Nature*-type plan that integrated goals linked to nature with local and national economic goals and flood control. Although the shift toward a more-integrated approach was triggered by a national policy initiative, Natura 2000 requirements clearly had a role in pushing the design toward a *Building with Nature* solution. The main conclusion of the research was that adopting a *Building with Nature* design is positively related to the successful implementation of Natura 2000 requirements in water infrastructure project subtypes similar to the cases studied. Specifically, applying a *Building with Nature* design contributed to successful project-level outcomes in Natura 2000 areas and, conversely, Natura 2000 requirements encouraged and enabled *Building with Nature* designs (Vikolainen 2012).

The research results thus pointed in a quite different direction from the initial hypothesis that Natura 2000 regulations would obstruct integrated *Building with Nature* developments. The emerging hypothesis was that, in practice, the goals of Natura 2000 regulations and the ideas behind *Building with Nature* are converging. The next section explains this finding in light of the learning process that took place at three governance levels.

## A Shift Toward Building with Nature: Governance Level

### Private Sector

Initially, public and private actors within the water infrastructure sector were not fully aware of the implications of Natura 2000 site designations, tried to downplay the negative effect of projects, or even ignored Natura 2000 requirements altogether. Examples of such attitudes are evident in some of the early cases (Southampton Dibden Bay, Western Scheldt Container Terminal, and Antwerp Deurganck Dock). After these developments were taken to court, with the outcome that the plans had to be rewritten to include a proper effects assessment, it became clear to project implementers that avoiding or ignoring Natura 2000 regulations was not the cheap option. This signified a change of approach toward including a greater environmental focus in projects, as is evident in later cases (Humber Estuary, Bremerhaven). Eventually, movements such as *Building with Nature*, *Working with Nature,* and *Flanders Bays* started to emerge as approaches that placed ecological goals at the start of the planning process (Oyster Reefs and Delfland Coast cases).

In terms of a theoretical approach used in the research, this learning process was attributed to a shift of project implementers’ perceptions. Initially, Natura 2000 was perceived as a threat, one that indeed led to the implementers’ economics-driven project plans bouncing off the “wall” of Natura 2000 procedures. Through a feedback loop, project implementers have learned that taking nature into account, alongside their economic motives, in project design increases the likelihood of a project being resistant to Natura 2000 restrictions. Even though placing ecological goals at the start of the planning process requires more resources in an early project stage, it can prevent possibly significant negative effects or at least allow them to be accounted for in a way acceptable to all stakeholders.

### EU Level

At the EU level, workable solutions were sought that would address the accumulated misunderstandings linked to Natura 2000 requirements and that would be acceptable to the member states, stakeholder organizations, and environmental NGOs. The solutions were discussed within the expert “Working Group on Estuaries and Coastal Zones” established by the European Commission. The consensus was that *Working with Nature* is the best way forward for all those involved, and this has been laid down in the “Guidelines on the implementation of the Birds and Habitats Directives in estuaries and coastal zones” (European Commission 2011). In these guidelines, the European Commission advocates the application of the *Working with Nature* approach in port development and dredging operations. It has recently been followed by a similar guideline for inland waterway development (European Commission 2012).

The strategy pursued by the European Commission, of encouraging best practices and models of good behavior, is argued in the Europeanization literature to be efficient in building technocratic legitimacy. The Commission cannot be accused of trying to impose “the view of Brussels” if it imitates a national policy model already in place somewhere in the EU that is perceived as the most successful (Radaelli 2000). Although *Building with Nature* is more of a project-level approach than a policy design, it has its roots in Dutch water management. In 2008, a commission appointed by the Dutch Government to address the long-term threats of climate change (the Delta Commission) recommended applying *Building with Nature* principles when replenishing beaches and the shoreline as the primary measure to guarantee the long-term safety and development of the coast (Delta Commission 2008). As such, advocating at the EU level for *Building with Nature*, albeit under the name *Working with Nature*, follows the path of imitating a perceived successful national approach.

### Member State Level

The case of the Kruibeke-Bazel-Rupelmonde flood control area showed how similar learning processes took place on the national and local levels in Belgium. At the national level, a large-scale water infrastructure project (Antwerp Harbor development) being implemented predominantly for its economic benefits was faced with the environmental requirements of Natura 2000. At the same time, local flood-defense projects were accorded low political priority. When the Antwerp Harbor development failed to meet the Natura 2000 requirements, a local flood control project was adjusted so that its design met compensation requirements so that both projects could be implemented with both nature and socioeconomic goals being fulfilled. As a result, the design of a local flood control area evolved toward balancing the flood defense, ecology, economic, and local stakeholder interests. At the local level, *Building* (or *Working*) *with Nature* effectively reconciled the previously conflicting interests and was acceptable to actors participating in the implementation including environmental groups, local farmer organizations and municipal residents. Similar regional-level experiences have been seen in the Netherlands (Warner et al. 2010, Wiering et al. 2010).

## A Shift Toward Building with Nature: Project Level

The data from the Harderwijk waterfront, the Zeewolde coastal zone, and the Kruibeke-Bazel-Rupelmonde flood control area case studies enabled identification of project-level variables that were influential in a successful implementation outcome. Besides *Building with Nature* design, four other variables were found: the size and borders of a Natura 2000 site; the conservation status and objectives of the Natura 2000 site; presentation of scientific data; and project administration.

The designation of a Natura 2000 site (SPAs and SACs) is always based on ecological data, and any decrease in their size is only allowed in exceptional cases (Case 57/89 Commission vs. Federal Republic of Germany 1991). The case studies showed that unwillingness of project implementers to designate or attempts to change the *size and borders of a Natura 2000 site,* were associated with negative implementation outcomes (project delays and cancelations). On the other hand, projects in which the implementing actors did not oppose the designation resulted in NGOs withdrawing their objections. As such, a proactive attitude toward the designation of SPAs and SACs (and the Natura 2000 framework in general) was a beneficial strategy for project implementers in the cases studied.

The *conservation status and objectives of a* *Natura 2000 site* were closely related to A*rticle 6 of the Habitats Directive*. The implementers of all the successful projects reported in Vikolainen et al. (2011) ensured that Natura 2000 conservation goals for a site would be achieved within their project designs, sometimes by establishing there would be no significant effect or by including a compensation project. That is, the case study results showed that successful projects adjusted their design to reflect Natura 2000 conservation objectives and contributed to their achievement. Conversely, less successful projects tried to “co-opt” new habitat based on existing nature development initiatives to neutralize the habitat that would be lost due to project work. On this basis, we conclude that a project design is more successful if it is fine-tuned to Natura 2000 conservation objectives. Hence, *the site’s conservation objectives* should feature alongside other project objectives as a starting point in any proposed development.

The case studies also demonstrated that *the presentation of scientific data* showing that a project’s plans ensured the coherence of the Natura 2000 network and enhanced the attainment of a site’s conservation objectives was positively related to successful project implementation. Such data are often part of an ecological effects assessment, either in the form of a pre-assessment or at another appropriate assessment stage. Sometimes, depending on a project’s size, this can form part of an Environmental Impact Assessment. The quasi-experimental comparison of cases (see Vikolainen et al. 2012) illustrated that there was some uncertainty in the assessment of each project’s ecological effects. It seemed, for a successful project outcome, that the presentation of the data was more important than its objective scientific certainty. Ecological scientific information always comes with uncertainty, and the actors’ interpretation of when the required level of certainty is achieved played a decisive role. Adopting integrated nature design, albeit indirectly, contributed to the confidence of actors in a project and to the required level of underpinning for a successful outcome. In this process, drawing on previous experience and using a consistent vocabulary, both in scientific reports and in written court defenses, proved useful. In the less successful case, the actors tried to use the full extent of available scientific knowledge to investigate the “significance” of a project’s effects but used the legal terminology inconsistently.

Similarly, the case studies showed that *project administration* was another factor that influenced the likelihood of a successful project implementation. The analysis showed that integrating socioeconomic and project goals requires a tailor-made approach, and that this is more easily achieved in a project administration that is flexible and can rapidly react to changing circumstances (Vikolainen et al. 2012). In less flexible and more traditional administrations, the development focus often shifts toward industrial and residential development needs and away from the nature development plans. As such, keeping people alert to what really matters in clearing potential regulatory hurdles related to Natura 2000 can be crucial for project implementation success.

The case study results reported in Vikolainen et al. (2013a) suggested that the first component of *Building with Nature design* that is the *integration* of nature and socioeconomic goals, was strongly related to a positive implementation outcome. In terms of the other components, the *integration* of nature and socioeconomic goals often coincided with *improved* nature values but less often with the *use* of nature dynamics. The link between *integration* and *improvement* is logical: to improve nature values, a project developer needs to first acknowledge and then integrate nature goals into a project design. The role of the *use* of nature dynamics is expected to increase as the knowledge gained in the *Building with Nature* research program becomes more widely available. A more thorough understanding of ecosystem dynamics and processes, and the application of this knowledge in practice, will boost the *use* of nature dynamics and thereby increase its role in achieving project outcomes. The scientific expertise gained related to the three design components will also constitute an added value of the *Building with Nature* approach compared to approaches that aim solely for integration.

## Guidance for Project Development in Natura 2000 Areas

The practical guidance for applying *Building with Nature* concepts in Natura 2000 areas proposed in this section builds on the above discussion of project-level variables. A habitat assessment under Article 6 is the main instrument of the Natura 2000 network approach, and this includes a number of steps that have been transposed into the national legislation of all EU member states. As such, it is a good starting point for a guidance document that outlines the “logic” involved in decision-making according to *Building with Nature* principles in the context of Natura 2000 areas. This guidance is meant for public and private actors intending to carry out a project and requires an awareness and general understanding of the context of the Natura 2000 policy and procedures in the various EU Member states (available in Vikolainen et al. 2013b).

There are at least two opportunities to introduce *Building with Nature* ideas into the Habitats Directive Article 6 procedure. The first is to introduce its elements in the pre-screening phase of a project, where *Building with Nature* concepts could be useful in avoiding significant adverse effects on a Natura 2000 area. The following questions could be helpful at this stage:

1. How can we adjust the *Building with Nature* design so that it contributes to Natura 2000 conservation objectives?
2. How can we make our *Building with Nature* initiative beneficial for the management of the Natura 2000 site?
3. How can we tailor the *Building with Nature* design to the size of and the effects on the Natura 2000 site (such as through a stepwise realization)?
4. How can we upscale or downscale the *Building with Nature* initiative to safeguard the overall coherence of the Natura 2000 network?

An outcome of such a reflection could be a *Building with Nature* design that supports the favorable conservation status of protected habitats and species.

The second opportunity to introduce *Building with Nature* becomes relevant if adjustments at the pre-screening phase have failed to produce a design without significant negative effects. This could occur with developments with an overriding economic interest (such as deepening a navigation channel or developing a port) that have to follow the procedure laid down under Article 6.4 of the Habitats Directive. If there is an absence of alternative solutions, possibilities could be explored to fulfill *Building with Nature* principles alongside, or as part of, a compensation plan to address the negative effects of project works on the conservation objectives of a Natura 2000 site. Here, the benefit of the *Building with Nature* concept is that it could generate local stakeholder support and/or create new possibilities for area development. It could provide a platform for cooperative interaction among the stakeholders and prevent frustrations and later legal contests, provided such interactions start as soon as it becomes apparent that compensation actions are unavoidable. The following questions could be helpful at this stage:

1. What are the possibilities for incorporating *Building with Nature* ideas as part of a compensation plan?
2. How can we adjust the *Building with Nature* design to benefit the interests of local stakeholders?
3. How can we use *Building with Nature* concepts to facilitate cooperative interaction among stakeholders?

**Fig. 1:**
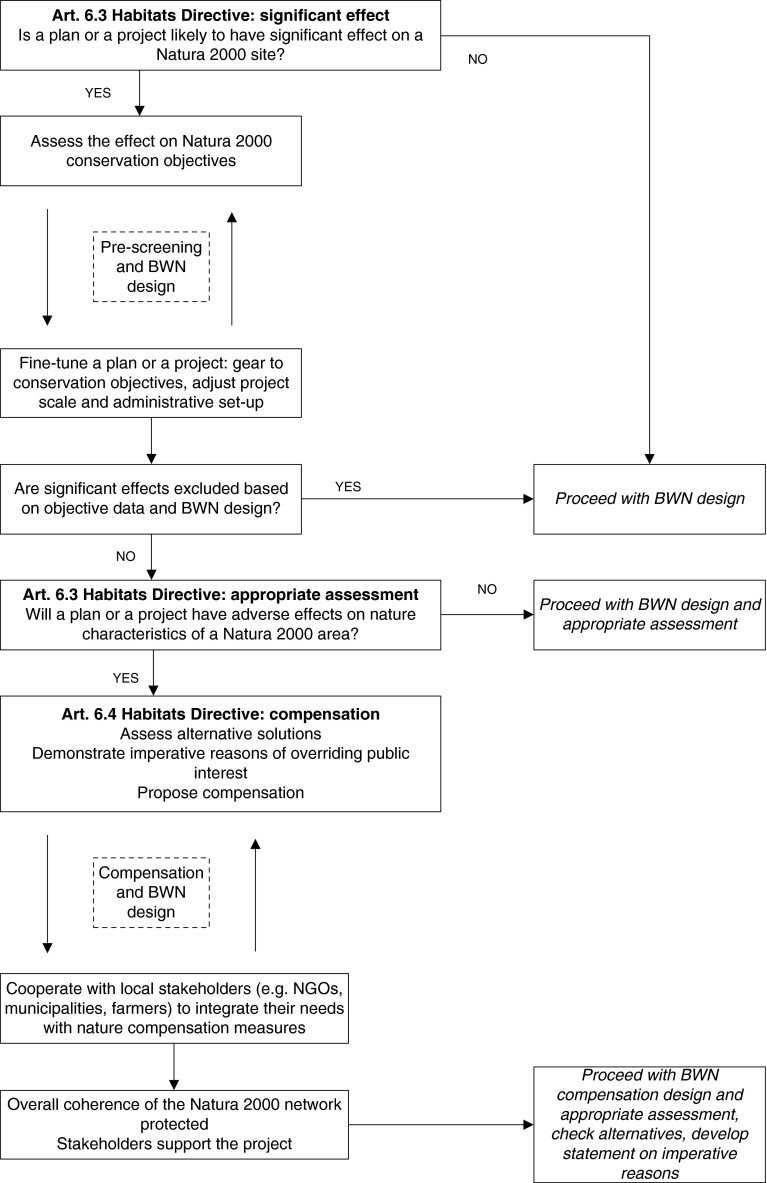
The *Building with Nature* design principles applied to the decision-making logic of Habitats Directive Assessment Article 6 (BWN = *Building with Nature*)

The flow chart in Fig. 1 summarizes the proposed decision-making logic in the context of an assessment according to Article 6 of the Habitats Directive.

## Discussion: Implications for the Dredging Industry

Initially, the dredging industry argued that a solution to regulatory bottlenecks would be to modify the Natura 2000 legislation to better fit with *Building with Nature* principles. However, our research found that Natura 2000 requirements actually provided opportunities to satisfy *Building with Nature* principles in the cases studied. Rather than attempting to modify the legislation, which would be a long-term strategy that would probably require examples of how *Building with Nature* attempts have been obstructed in the current regulatory setting, a project developer could choose to proactively work with the legislation. Such a strategy requires a perceptual change: from seeing regulations as “barriers” to *Building with Nature* to viewing regulations as “opportunities” for *Building with Nature*.

The strategy of proactively working with the legislation could be applied in other governance contexts where there are similar overarching nature regulations as Natura 2000. However, one should be aware that legal barriers outside the EU may be less restrictive than those within the EU. North-west European estuaries and coastal zones are densely populated areas that experience the combined pressure of economic activities and conservation objectives. In places where environmental regulations are less stringent, there may be no pressure to innovate or pilot new methods in designing infrastructure. For instance, in a study based in Virginia Beach (USA), Stevens et al. (2013) found that local stakeholders would probably continue to support existing water engineering practices, until there was stronger pressure from regulatory agencies or from budget constraints to find other innovative ways.

In the absence of regulatory pressure, *Building with Nature* is likely to spread as a consequence of the dynamics underlying the adoption of best practices by supranational authorities and transnational actors. According to Tews et al. (2003), international environmental agreements and aspirational recommendations often reflect the “high” environmental standards of pioneering countries and the agenda-setting power of ambitious, well-organized private actors from those countries. National adoption of policy innovations practiced in other countries, or modeled on internationally promoted “best practices,” is also facilitated by non-state actors. In the case of *Working with Nature*, the World Association for Waterborne Transport Infrastructure (PIANC) fulfilled this role (PIANC 2011). Further, once new approaches to environmental policy are practiced in “forerunner” countries, it becomes increasingly difficult for other countries to resist adopting the same approach without threatening their image as legitimate members of an environmentally responsible global society. As a result, national environmental policies tend to converge on the level established in “forerunner” countries (Tews et al. 2003). As a consequence, despite the weak enforcement mechanism, *Building*, or *Working, with Nature* stands a good chance of becoming a best practice in estuary and coastal zone water management outside Europe.

There is a wide and growing appreciation among politicians, policymakers, and stakeholders that sustainable ways of designing water infrastructure are needed. For example, there is a growing interest in applying large-scale sand replenishment schemes, similar to that executed on the Dutch coast, in Peru (Lima), Vietnam (Da Nang), UK (Lincolnshire and Suffolk), and Sweden (Ystad). The feasibility of large-scale sand replenishment in these locations will be researched in the NatureCoast program funded by the Dutch Science and Technology Foundation (Technologiestichting STW, 2013).

## Conclusions

The research question addressed in this article was: what are the implications of the European nature conservation policy for project developers intending to apply *Building with Nature* in or near Natura 2000 areas? The project-level variables identified in the course of the research have been used to propose guidance for a *Building with Nature* development involving dredging and port development in Natura 2000 areas. The research results suggest that to increase the likelihood of successfully implementing a water infrastructure project, an efficient business strategy would be to work proactively with the legislation in line with the decision-making logic indicated in the provided guidance. While this requires more resources in the early stages of a project, it avoids potential negative environmental effects or enables them to be accommodated in a way that is acceptable to all stakeholders. Despite the apparently weak enforcement mechanism, *Building with Nature* has the potential to become an international water management best practice.
